# Evaluation of peri-implant soft and hard tissues behavior in screw-retained crowns by the biologically oriented preparation technique (BOPT): Ambispective longitudinal analytical study

**DOI:** 10.4317/jced.58924

**Published:** 2022-01-01

**Authors:** Victoria Mandillo-Alonso, Rocío Cascos-Sánchez, José-Luis Antonaya-Martín, Martín Laguna-Martos

**Affiliations:** 1 DDS, MDent. Collaborating Professor of Master Prótesis sobre Implantes. Rey Juan Carlos University. Avenida de Atenas s/n, 28922, Alcorcón, Madrid, España; 2 DDS, MSD, MDS. MDent. Collaborating Professor of Postgrade Implantoprótesis Avanzada. Complutense University. Plaza Ramón y Cajal s/n, 28040, Madrid, España; 3 DDS, MSD, MDS. MDent. Collaborating Professor of Master Prótesis sobre Implantes. Rey Juan Carlos University. Avenida de Atenas s/n, 28922, Alcorcón, Madrid, España; 4 DDS, MSD, PhD, MDent. Director of Master Prótesis sobre Implantes. Rey Juan Carlos University. Avenida de Atenas s/n, 28922, Alcorcón, Madrid, España; 5 DDS, MSD, PhD, MDent. Collaborating Professor of Postgrade Implantoprótesis. Complutense University. Plaza Ramón y Cajal s/n, 28040, Madrid, España; 6 DDS. Collaborating Professor of Master Prótesis sobre Implantes. Rey Juan Carlos University. Avenida de Atenas s/n, 28922, Alcorcón, Madrid, España

## Abstract

**Background:**

Clinical and radiographic evaluation of soft and hard tissues around convergent collar implants and shoulderless abutments.

**Material and Methods:**

Ambispective longitudinal analytical study with a sample size of 32 implants in 21 patients treated in a private dental clinic. Patients were divided into two groups: Prama Implants or group 1 (n=21) and Shelta implants combined with XA abutment or group 2 (n=11). Probing depth, horizontal mucosa thickness, peri-implant bone loss, plaque and bleeding after one-and two-year follow up are analyzed.

**Results:**

In group 1, mean probing depth value was 1.67 mm (±0.58) and mean horizontal mucosa thickness value was 2.71 (±0.96). In group 2 mean probing depth was 2.18 (±0.40) and mean horizontal mucosa thickness value was 3.27 mm (±1.19). In group 1 an 85.7% of peri-implant bone level was maintained and a 14.3% increased. In group 2 a 100% of peri-implant bone level was maintained. In group 1 a 19% presented plaque when crowns were removed and in group 2 a 18.2% presented plaque. Neither of two groups presented spontaneous bleeding when crowns were removed. A 52.4% presented probing bleeding in group 1 and a 45.4% in group 2.

**Conclusions:**

Biologically guided crowns design seems to provide peri-implant hard and soft tissue stability.

** Key words:**Biologic width, peri-implant soft tissue, marginal bone loss, transmucosal implant, convergent collar, BOPT (biological oriented preparation technique), BOPT abutment, soft tissue stability.

## Introduction

Dental implants are currently considered an effective treatment for functional and aesthetic rehabilitation of missing teeth. Implant treatment success is determined by the integration and stabilization of hard and soft tissues (1,2). Osseointegration was defined by Branemark in 1969 as the “direct functional and structural connection between healthy bone and a surface of an implant under load” (1). On the other hand, the stability of peri-implant soft tissues gives a natural appearance to rehabilitation while protecting it from external agents and avoiding bone resorption (3).

Recently, new implant abutments and crown designs have been developed in order to improve insertion of peri-implant soft tissues to avoid bacterial contamination of the alveolar bone (3). These designs are inspired by the Biological Oriented Preparation Technique (BOPT) described by Ignazio Loi in 2008 (4).

This technique is based on a vertically prepared prosthodontic protocol with no finish line which allows the mucosa adaptation to the prosthetic profile determined by the crown (4). Thus, by modifying the contours of the crown, the clinician can control and modify the marginal level of peri-implant soft tissues. On natural teeth, preparation eliminates the anatomical cement-enamel junction (CEJ) and places the termination line on the crown, not on the tooth. This allows it to create an ideal gingival architecture modulating the emerging profile of the crowns. Same principle could be applied in intramucosal implants restorations with convergent neck and abutment designs, whose objective is to maximize the available space for the soft tissues. A convergent profile allows tissue to migrate coronally to the area of smaller diameter in early stages of healing, creating a thick, stable and more coronal connective seal, below the profile created with the restoration. This sets up a protective barrier for soft tissue and peri-implant structures. (3-10).

Two areas are defined in this technique: Booster area (BO) or tissue enhancer zone and Prop Tissue up area (PT) or supporting zone of the gingival margin. BO is defined by the convergence of the cervical area of the tooth, implant or abutment and enhances the thickening and coronal tissue migration. PT belongs to the crown and its functions are to maintain the gingival margin to prevent collapse and to stop coronal migration of the gingival margin. The slight over-contouring that characterizes BOPT technique delimits a negative pressure area formed by the crown, the lip and the gingival margin. This, together with mechanotransduction phenomenon helps horizontal thickening of soft tissues over the course of the patient’s life (11).

The main objective of this research was the clinical and radiographic evaluation of peri-implant soft and hard tissues around Prama implants (Sweden Martina®) and XA abutments (Sweden Martina®) after one- and two-year follow up. The null hypothesis was that BOPT technique does not result in a sTable and protective peri-implant mucosa seal and sTable peri-implant bone level.

## Material and Methods

-Study design and patient selection.

A preliminary ambispective longitudinal analytical study was carried out from April 2017 until September 2020 at a private dental clinic (Instituto Manchego de Implantología y Estética, Alcázar de San Juan, Spain). The study sample consisted of 21 patients (12 women and 9 men) susceptible to implant treatment took part in the research. The average follow-up time was 16 months.

This study has been approved by the Research Ethics Committee (CEI) of the University Rey Juan Carlos de Madrid, with registration number 1510202018220, following the recommendations of the Declaration of Helsinki. All patients were informed of the purpose and characteristics of the study and signed an informed consent after reading it and resolving any pertinent doubts.

Participants were included in the study according to the following inclusion criteria: patients susceptible to implant treatment, patients over 18 years of age, patients treated with Prama (Sweden Martina®) or Shelta implants (Sweden Martina®), patients with a minimum of one year follow-up, patients who have good oral hygiene and motivated to maintain it, single and partial rehabilitations, anterior and posterior area rehabilitations. On the other hand, the exclusion criteria were the following: patients with medical and dental history that make it difficult to place implants, patients with diseases that may affect bone metabolism such as arthritis or osteoporosis, patients with systemic diseases not controlled or polymedicated, smokers of more than ten cigarettes a day and patients with metal allergies.

A clinical and a radiographical study were carried out. Patients were divided into two groups according to the type of implant that has been placed: Prama implants or group 1 (n=21) and Shelta implants or group 2 (n=11). Shelta implants were combined with a convergent intermediate XA abutment (Sweden Martina®).

The following variables were collected: probing depth, horizontal mucosa thickness, peri-implant bone level, plaque, spontaneous bleeding, probing bleeding, sex, age, implant position, diameter of the implant, implant length, implant type, presence of connective tissue graft, presence of xenograft, antagonist, immediate implant, intermediate abutment, abutment intermediate size and follow-up time.

-Surgical procedure and post-operative care.

All patients were treated by the same operator, M.L.M. Shelta implants placed in the anterior esthetic sector were accompanied by a connective tissue graft (CTG) and bone regeneration therapy with xenograft (Bio-Oss®, Geistlich Pharma AG) in the gap. Prama implants were placed in the posterior sector and no regenerative therapy was performed. All Prama implants were placed in a bone level position in order to let the convergent neck to the soft tissue. All Shelta implants were placed in a subcrestal position (1-2 mm). All the implants were placed with a minimum insertion torque of 30 N.

In type I and II sockets, tooth extraction and implant placement were carried out in the same surgical act, that is, “immediate implant placement”.

Patients were medicated with Amoxicillin/clavulanic 875/125 mg one dose every 8 hours for a week and with ibuprofen 600 mg one dose every 8 hours in case of pain. In addition, after the first 24 hours, they were prescribed a 0.2% chlorhexidine rinse for a week at night.

After surgery, all patients attended a review at one week, one month and, finally, at three or five months in order to take impressions for the definitive crown. Average osseointegration time was 5 months.

-Restorative treatment.

Prama implants were rehabilitated with a customized, screwed healing cap the same day of surgery (A-MPSCI-330-EX, Sweden Martina®). The customized immediate healing cap was made with flowable composite following socket anatomy. Shelta implants were rehabilitated with a screw-retained immediate aesthetic provisional (SH-CTABU-F-380, Sweden Martina®) made from a previous wax-up. The objective of provisionalizing the same day of the surgery was to preserve the clot stability at the same time that the soft tissue healed according to the shape of the provisional.

**Figure 1:**
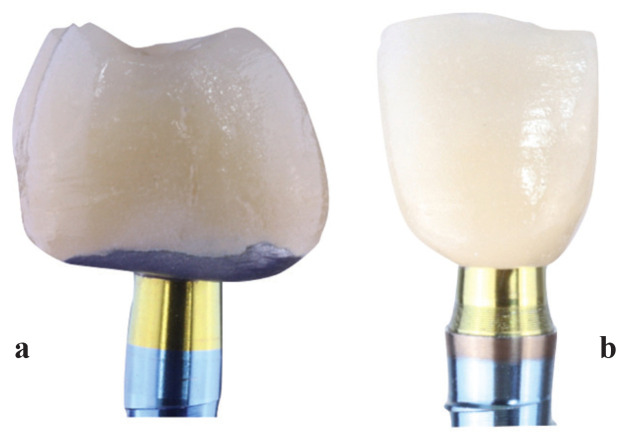
a. Prama restoration. b. Shelta-XA abutment restoration.

Finally, after osseointegration time and soft tissue modeling, digital impressions were taken using a 3Shape TRIOS® intraoral scanner to make definitive prosthesis. All crowns were screw-retained implant supported made with milled Cr-Co metal and feldespathic ceramic following a BOPT design, which is 1 or 1.5 mm below the gingival margin in order to simulate the natural emergence profile of the teeth. Crowns were made by CAD/CAM design software. In posterior area, all crowns embrace 0.8 mm of the Prama implant convergent neck to increase stability (Fig. 1a). Shelta implant and XA abutment are represented in Figure 1b. Screw access channel were covered by teflon and composite.

-Data collection.

Data collected was: clinical history; initial frontal, lateral and occlusal intraoral photographs; photographs with the crowns removed in order to evaluate signs of inflammation or bleeding; peri-implant horizontal sulcus length measurement; vertical depth probing measurement; and, finally, initial and final intraoral radiographs. Data was collected by a single operator, V.M.A. except the initial intraoral radiographs which were carried out by M.L.M, second operator.

-Soft tissue evaluation.

**Figure 2:**
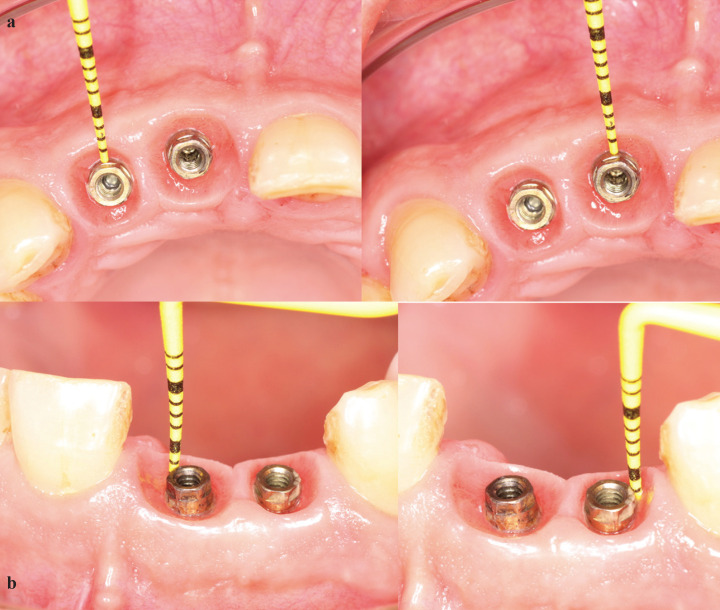
a. Periodontal probe placed transversally from the implant platform/abutment to the end of the horizontal gingival sulcus. b. Periodontal probe placed vertically to evaluate the mucosal seal around XA abutment and around Prama convergent neck.

Clinically measurements were made with a PCVUNC12PT periodontal probe (Hu-Friedy®), placing it transversally from the implant platform or abutment to the end of the horizontal gingival sulcus (Fig. 2a). Measurements were also made in a vertically direction (Fig. 2b) to evaluate the mucosal seal around XA abutment and around Prama convergent neck.

-Hard tissue evaluation.

Marginal bone level was evaluated on intraoral radiographs. Radiographs made the day of definitive crown placement and follow-up appointment were collected for the present study. All radiographs were performed with a Super-bite positioner (Kerr Hawe®) and with PSPIX phosphor plates (Acteon®). In this study radiographs are used to determine if there was gain, loss or maintenance of marginal bone level.

-Statistical analysis of the data.

Statistical analysis was carried out using the SPSS Statistics version 25 software application (IBM; Armonk. NY, USA), using the Student’s t-test for repeated measures in the contrast of variables between the initial and control averages and between groups of cases independent from each other.

## Results

The study sample consisted of 32 implants. Table 1 summarizes the characteristics of the implants. Implants were classified in two groups: group 1 (n=21) included Prama implants, without connective tissue graft and without xenograft, and group 2 (n=11) included Shelta implants, with connective tissue graft and with xenograft. All Shelta implants were combined with XA abutments: 4 mm-abutment in 36.4%, 5 mm-abutment in 45.5% and 6 mm-abutment in 18.2%.

**Table 1:**
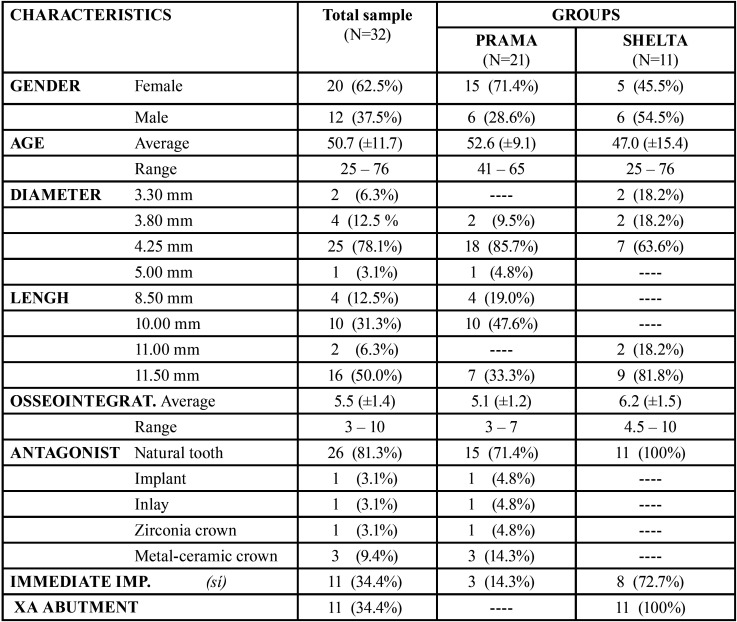
Descriptive analysis. Sample characteristics.

A total of 20 implants (62.5%) were placed in women and 12 implants (37.5%) were placed in men. The age of the patients ranged between 25 and 76 years old with an average age of 51 years (±11.7). In this sample, 11 implants (34.4%) were placed in the anterior area (incisors or canines) and 21 implants (65.5%) were placed in the posterior area (molars or premolars). Osseointegration time ranged between 3 and 10 months, with an average time of 5.5 months (± 1.4). Most of implant antagonists were natural pieces (81.3%). 18.7% of antagonists were implants, inlays, zirconia crowns or metal-ceramic crowns. 34.4% of the implants were placed immediately after extraction. The mean follow-up time was 16.4 (± 5.47) months.

Most of the implants showed a probing depth value of 2 mm (65.5%), with a range between 1 and 3 mm (mean of 1.84 mm). About horizontal mucosa thickness in most of the cases the value observed was between 1 and 5 mm (mean 2.91 mm). In a 90.6% of the cases peri-implant bone level was maintained. 81.3% of the implants had no plaque. There was no any implant with spontaneous bleeding, however there was probing bleeding in a 50% of the cases. These outcome variables are reflected in Table 2.

**Table 2:**
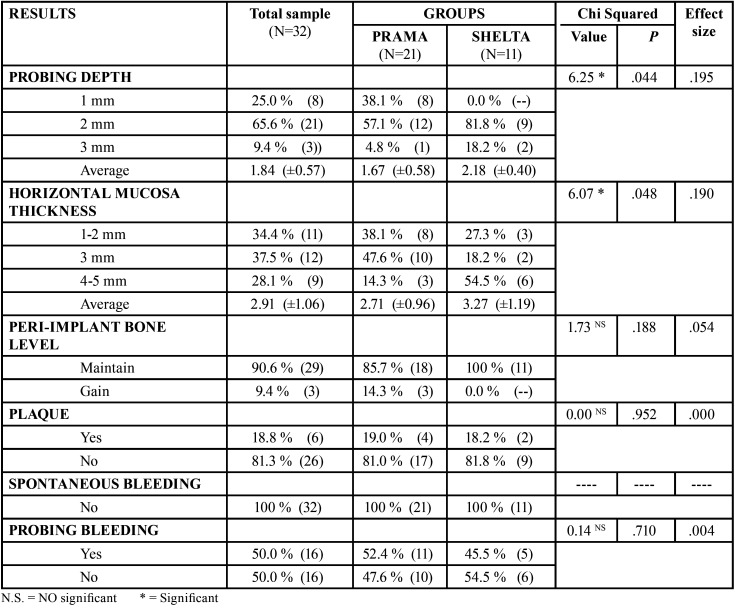
Descriptive and comparative analysis.

In Shelta implants probing depth was greater (2.18 mm) than in Prama implants (1.67 mm). In Shelta group horizontal mucosa thickness was greater (3.27 mm) than in Prama implants (2.71). It can be concluded that there is a statistically significant difference (*p*=0.044; *p*=0.048) in both variables.

-Effect of factors on peri-implant soft and hard tissues.

•Gender.

In group 1 horizontal mucosa thickness in women (2.93 mm) was greater than in men (2.17 mm). Probing depth in women (2.40 mm) was greater than in men (2 mm) in group 2. Peri-implant bone level and plaque does not reach statistical significance (*p*=0.0876; *p*=0.0815).

•Age.

Horizontal mucosa thickness was greater (*p*<0.01) in patients younger than 52 years. Probing depth, peri-implant bone level, plaque and probing bleeding does not reach statistical significance (*p*=0.620; *p*=0.471; *p*=0.865; *p*=0.723).

•Implant position.

Horizontal mucosa thickness, peri-implant bone level, plaque and bleeding does not reach statistical significance (*p*=0.160; *p*=0.188; *p*=0.952; *p*=0.710). In Shelta implants probing depth is greater (2.18 mm) than in Prama implants (1.67 mm).

•Immediate implant placement.

In Prama group probing depth was greater (1.72 mm vs 1.32 mm) when immediate implant was not placed. In Shelta group horizontal mucosa thickness was greater (4 mm vs 3 mm) when immediate implant was not placed. However there is not statistical significance (*p*=0.619; *p*=0.723) to considered as evidence of association.

## Discussion

Predictability and success of implant rehabilitation treatments are associated with the presence of a good soft tissue thickness. New implant designs, abutments and crowns inspired by the BOPT on teeth improve insertion of the peri-implant soft tissues in order to avoid bacterial contamination. We know that connective tissue forms a protective barrier around implants or intermediate abutments. When connective tissue stabilizes, it prevents apical migration of the junctional epithelium and determines the amount of bone resorption. Therefore, it acts as a sealing barrier, like a ring, providing better resistance to mechanical and bacterial aggressions (12). The objective of this research was to evaluate the clinical and radiographic peri-implant soft and hard tissue outcomes. The null hypothesis was that BOPT technique does not result in a stable and protective peri-implant mucosa seal and stable peri-implant bone level.

According to Vela *et al*. (13) the more connective tissue there is, the shorter the junctional epithelium will be, and the less peri-implant bone is reabsorbed. In 2016, they evaluated the orientation of collagen fibers around platform switching implants obtaining revealing results in which the length of the sulcus was shorter than in similar histological studies done in humans, and bone resorption was minimal compared to similar radiological studies. The mean sulcus length was 1.02 mm. 81% of the thickness of the connective tissue was maintained thanks to the conical shape of the abutments, which allow a greater soft tissue thickness. They concluded that connective tissue is crucial to stabilize the apical migration of the junctional epithelium and, consequently, to decrease bone resorption. Circular fibers of connective tissue could be a key factor in soft tissue stabilization around implants and consequently, could protect peri-implant bone better.

**Figure 3:**
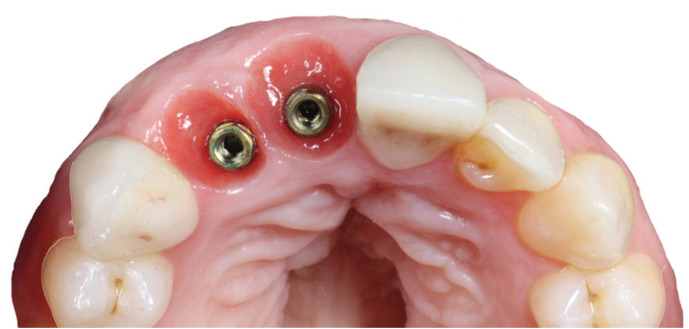
Horizontal everted “short sulcus”.

In this study, when performing BOPT philosophy was performed, we obtained a horizontal everted “short sulcus” (Fig. 3) and supracrestal connective tissue around the convergent Prama neck or the XA abutment. What we pursue is to obtain a stable mucosal seal in order to protect our implants. It was obtained an average probing depth of 1.67 mm in Prama implants, which indicates a stable and protective connective tissue, there is a good mucosa seal and our probe did not penetrate beyond this limit. In Shelta implants with XA abutment, the probing depth measurement was 2.18 mm. In XA abutment designs, a greater depth could be expected because there is a greater distance between the implant platform and the most coronal area of soft tissue than in Prama implants, that is, there is more connective tissue length when XA abutments are used. For this reason, a mean of 2.18 mm is considered optimal and indicates good peri-implant mucosal sealing.

Peri-implant mucosa thickness (MT) is the horizontal dimension of the peri-implant soft tissue and especially in the coronal area. MT has an important role in peri-implant health and in functional and aesthetic results of implant therapy. Thin soft tissues can cause crestal bone loss during the formation of the peri-implant sealing, which established that we need a minimum of 2 mm of peri-implant mucosa. In this way, predictable long-term functional and aesthetic results are achieved, and marginal bone loss and mucosal recession is minimized. The following categorization is proposed: thin peri-implant mucosa (<2 mm) and thick peri-implant mucosa (≥2 mm) (14). In our study we can observe a Prama average horizontal mucosa thickness of 2.71 mm (±0.96) and a Shelta average of 3.27 mm (±1.19). However, we need a larger sample in order to obtain more significant results.

According to these results the null hypothesis that BOPT technique does not result in a sTable and protective peri-implant mucosa seal and stable peri-implant bone level was rejected.

Agustín *et al*. (9) carried out a study to evaluate the behavior of the soft tissue around conventional screw-retained crowns, conventional cemented crowns and BOPT-type cemented crowns. They conclude saying that BOPT cemented crowns obtain better keratinized gingiva, less probing depth and lower incidence of probing bleeding than screw-retained crowns or conventional cemented crowns. In addition, there is a direct correlation between soft tissue and marginal bone loss: the better the keratinized gingiva is, the less the marginal bone loss, the less probing depth and the less bone loss there is. Rompen *et al*. (15) affirm that the use of concave transmucosal profiles seems to allow a better and more predicTable soft tissue stability in esthetic areas than divergent profiles.

Regarding marginal bone loss, in this study a 85.7% of Prama implants maintained peri-implant bone level and a 14.3% gained. A 100% of Shelta implants maintained peri-implant bone level. Canullo *et al*. (16) say the use of a BOPT protocol with convergent tissue level neck implants maintains hard tissues stability after 3 years of follow-up. Agustín *et al*. (8) compared marginal bone loss in three types of implant-supported crowns after 3 years of functional loading. They analyzed conventional screw-retained crowns, conventional cemented crowns, and BOPT-type cemented crowns and conclude that BOPT cemented crowns suffer the least marginal bone loss. The same authors affirm in another study (17) that implants with convergent neck designs have less marginal bone loss compared to implants with divergent neck designs.

It is worth noting the role of the abutment height. A study carried out by Marconcini *et al*. (18) declare that implants with convergent necks present an excellent preservation of marginal bone after one year from the definitive prosthetic restoration. Minimal resorption was found with abutments higher than 5 mm. Galindo-Moreno *et al*. (19) assessed marginal bone loss around 0.5 mm, 1 mm, 2 mm and 4 mm intermediate abutments and conclude saying that there is greater marginal bone loss in short abutments. Spinato *et al*. (20) say the lower the abutment height is, the greater the marginal bone loss is.

Hermann *et al*. (21) indicate that marginal bone loss around two-piece implants is significantly higher than in one-piece implants. However, they connected the restoration directly to the implant, without intermediate abutments. It is also important to notice, as is indicated by Bressan *et al*. (22) and Galindo-Moreno *et al*. (19) in their studies, that repeated connections and disconnections usually performed on implant head restorations result in a in bone loss increase of 0.43 mm. The use of one abutment one time concept preserves biological width and minimizes marginal bone loss.

The present study had some limitations. Despite these significant results that directly report soft and hard tissue peri-implant stability with the biologically oriented preparation technique, it is necessary to clarify that the sample size is small and the follow-up time is short. Also, it would be interesting to perform histological studies that can guide us on the junctional epithelium and connective tissue lengths. This study includes only clinical results.

In conclusion, convergent neck implants and shoulderless abutments in combination with a BOPT restoration result in a stable and protective peri-implant mucosa seal and stable peri-implant bone level. However, further studies are needed to support the findings of the present study. It is necessary to extend the sample size and the follow-up time in order to obtain more significant results.
